# Low-dose betamethasone-acetate for fetal lung maturation in preterm sheep

**DOI:** 10.1016/j.ajog.2017.11.560

**Published:** 2018-01

**Authors:** Augusto F. Schmidt, Matthew W. Kemp, Judith Rittenschober-Böhm, Paranthaman S. Kannan, Haruo Usuda, Masatoshi Saito, Lucy Furfaro, Shimpei Watanabe, Sarah Stock, Boris W. Kramer, John P. Newnham, Suhas G. Kallapur, Alan H. Jobe

**Affiliations:** 1 Division of Neonatology and Pulmonary Biology, Cincinnati Children’s Hospital Medical Center, Cincinnati, OH; 2 School Women’s and Infants’ Health, University of Western Australia, Perth, Australia; 3 Division of Neonatology, Pediatric Intensive Care and Neuropediatrics, Medical University Vienna, Vienna, Austria; 4 Department of Obstetrics and Gynecology, Tohoku University, Sendai, Japan; 5 MRC Centre for Reproductive Health, University of Edinburgh Queen’s Medical Research Institute, Edinburgh, United Kingdom; 6 Maastricht University Medical Center, Maastricht, Netherlands

**Keywords:** antenatal corticosteroids, betamethasone, fetal lung maturation, prematurity

## Abstract

**Background:**

Antenatal steroids are standard of care for women who are at risk of preterm delivery; however, antenatal steroid dosing and formulation have not been evaluated adequately. The standard clinical 2-dose treatment with betamethasone-acetate+betamethasone-phosphate is more effective than 2 doses of betamethasone-phosphate for the induction of lung maturation in preterm fetal sheep. We hypothesized that the slowly released betamethasone-acetate component induces similar lung maturation to betamethasone-phosphate+betamethasone-acetate with decreased dose and fetal exposure.

**Objective:**

The purpose of this study was to investigate pharmacokinetics and fetal lung maturation of antenatal betamethasone-acetate in preterm fetal sheep.

**Study Design:**

Groups of 10 singleton-pregnant ewes received 1 or 2 intramuscular doses 24 hours apart of 0.25 mg/kg/dose of betamethasone-phosphate+betamethasone-acetate (the standard of care dose) or 1 intramuscular dose of 0.5 mg/kg, 0.25 mg/kg, or 0.125 mg/kg of betamethasone-acetate. Fetuses were delivered 48 hours after the first injection at 122 days of gestation (80% of term) and ventilated for 30 minutes, with ventilator settings, compliance, vital signs, and blood gas measurements recorded every 10 minutes. After ventilation, we measured static lung pressure-volume curves and sampled the lungs for messenger RNA measurements. Other groups of pregnant ewes and fetuses were catheterized and treated with intramuscular injections of betamethasone-phosphate 0.125 mg/kg, betamethasone-acetate 0.125 mg/kg, or betamethasone-acetate 0.5 mg/kg. Maternal and fetal betamethasone concentrations in plasma were measured for 24 hours.

**Results:**

All betamethasone-treated groups had increased messenger RNA expression of surfactant proteins A, B, and C, ATP-binding cassette subfamily A member 3, and aquaporin-5 compared with control animals. Treatment with 1 dose of intramuscular betamethasone-acetate 0.125mg/kg improved dynamic and static lung compliance, gas exchange, and ventilation efficiency similarly to the standard treatment of 2 doses of 0.25 m/kg of betamethasone-acetate+betamethasone-phosphate. Betamethasone-acetate 0.125 mg/kg resulted in lower maternal and fetal peak plasma concentrations and decreased fetal exposure to betamethasone compared with betamethasone-phosphate 0.125 mg/kg.

**Conclusion:**

A single dose of betamethasone-acetate results in similar fetal lung maturation as the 2-dose clinical formulation of betamethasone-phosphate+betamethasone-acetate with decreased fetal exposure to betamethasone. A lower dose of betamethasone-acetate may be an effective alternative to induce fetal lung maturation with less risk to the fetus.

Antenatal corticosteroids (ANS) are a life-saving therapy for premature infants. Despite the well-documented effectiveness and widespread use, questions remain regarding formulation, dosing, route of administration, and repeated doses^1^ because the ANS used for fetal lung maturation has not been evaluated rigorously.^2^ In fact, multiple treatments are used around the world based on drug availability and historical use without experimental or clinical evaluation.^3^ The most commonly recommended and tested regimens are intramuscular dexamethasone-phosphate 6 mg every 6 hours for 4 doses (total dose 24 mg) or the combination of betamethasone-acetate (Beta-Ac) and betamethasone-phosphate (Beta-P) 12 mg every 24 hours for 2 doses.^4^, ^5^, ^6^ The maturational effects of ANS likely are determined by the length and amplitude of fetal exposure. Although several formulations are used interchangeably, the pharmacokinetics profiles of the formulations are different and may not all be equally effective.

The 2-dose regimen of Beta-Ac+Beta-P was used by Liggins and Howie^7^ in their seminal trial and has been adopted as the standard therapy for most randomized controlled trials.^8^ The Beta-P component is dephosphorylated rapidly to the active drug, which, in the sheep model, results in an early maternal peak concentration of approximately 130 ng/mL at <1 hour, with a half-life of 4 hours.^9^, ^10^ The microparticulate Beta-Ac component is slowly deacetylated, which results in a later peak and more prolonged half-life compared with the phosphate component. The clinical combination of the 2 drugs results in a complex pharmacokinetics with a half-life of 14 hours.^10^ Drug levels in humans are limited to paired maternal and cord blood fetal samples that are collected from deliveries shortly after an ANS treatment. An estimate of the initial half-life in maternal plasma was 9 hours with peak maternal levels of betamethasone at 3–4 hours of approximately 60 ng/mL and fetal plasma beta levels of approximately 30% of maternal levels.^11^ In contrast, in preterm sheep a single Beta-Ac dose of 0.25 mg/kg promotes fetal lung maturation with a fetal drug level of approximately 2 ng/mL at 24 hours.^9^

The preterm sheep model has been used widely for studies of fetal lung maturation because the fetus can be catheterized, the gestation length is appropriate for testing exposure to delivery intervals relevant to the human, and physiological responses can be evaluated. Using a preterm fetal lamb model, we showed that maternal intramuscular Beta-Ac+Beta-P was more effective than Beta-P alone for improving lung compliance and increased expression of surfactant protein mRNA.^12^ Low-dose maternal infusions of Beta-P in catheterized pregnant sheep also increased expression of fetal lung maturation markers, despite much lower fetal maximal concentrations than the Beta-Ac+Beta-P that is used clinically.^13^ The pharmacokinetic data suggest that the duration of fetal exposure to a low plasma level of corticosteroids may promote fetal lung maturation better than shorter exposures to the higher plasma concentrations.

Hence, considering the pharmacokinetic profile of Beta-Ac, we hypothesized that a single lower dose of Beta-Ac would promote lung maturation comparable with the standard treatment with lower fetal exposure. Clinical studies of drug and dosage are impractical for either pharmacokinetic or pharmacodynamic assessments of new treatment strategies. Therefore, we tested 3 doses of Beta-Ac that were equivalent to the full, one-half, and one-quarter of the total Beta-Ac+Beta-P dose in preterm fetal and newborn lamb models as an initial strategy to refine a treatment strategy for clinical evaluation.

## Methods

### Animal studies

The protocols were approved by the animal ethics committee of The University of Western Australia (RA/3/100/1378). We used chronically catheterized pregnant sheep and their fetuses for drug level measurements and separate preterm ventilated lambs to assess fetal lung maturation after maternal ANS treatment. To reduce the risk of preterm labor from ANS, time-mated ewes with singleton fetuses were treated with 1 intramuscular dose of 150-mg medroxyprogesterone acetate (Depo-Provera; Pfizer, New York, NY) at 110 days of gestation for pharmacokinetics studies and at 115 days of gestation for pharmacodynamics studies. No other doses of medroxyprogesterone acetate were given, and we did not administer other tocolytics.

**Figure 1:**
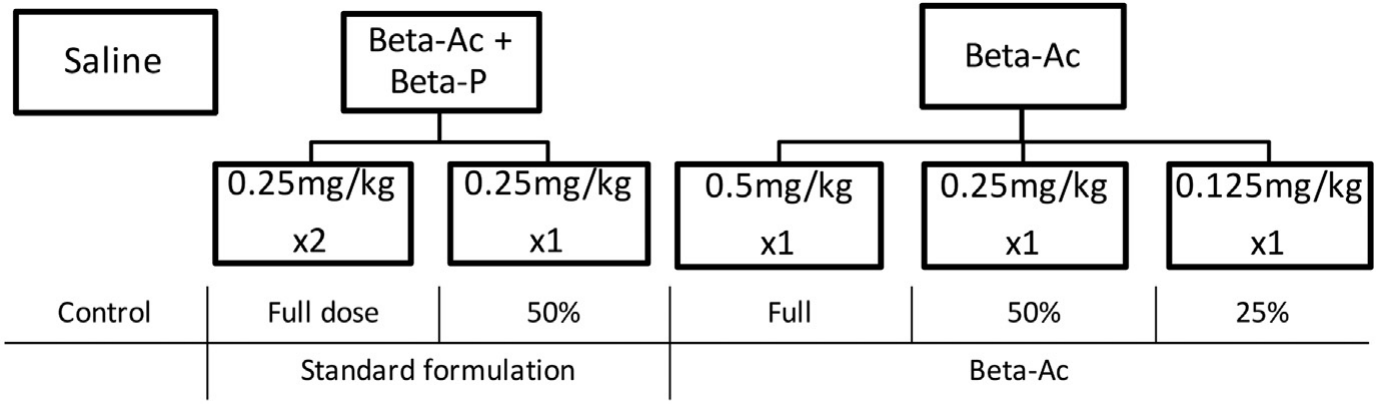
Experimental design Negative control animals were treated with intramuscular saline solution. A group of animals was treated with the clinically used formulation of Betamethasone-acetate + Betamethasone-phosphate as either 2 doses of 0.25 mg/kg 24 hours apart (clinical dose) or a single dose (50% total clinical dose). Another group of animals was treated with Betamethasone-acetate only either with a dose equivalent to the full clinical dose (0.5 mg/kg), 50% of the clinical dose (0.25 mg/kg), or 25% of the clinical dose (0.125 mg/kg). *Beta-Ac+Beta-P*, betamethasone-acetate+betamethasone-phosphate. *Schmidt et al. Betamethasone-acetate for fetal lung maturation. Am J Obstet Gynecol 2018*.

For pharmacodynamics studies, animals were randomized to receive either saline solution (control animals) or 1 of the following treatments: 2 doses of Beta-Ac+Beta-P (Celestone Chronodose; gift from Merck Shar & Dohm Corp., Inc, Kenilworth, NJ) 0.25 mg/kg intramuscularly 24 hours apart, 1 dose of Beta-Ac+Beta-P of 0.25 mg/kg intramuscularly, 1 dose of Beta-Ac 0.5 mg/kg intramuscularly, 1 dose of Beta-Ac 0.25 mg/kg intramuscularly, or 1 dose of Beta-Ac 0.125 mg/kg intramuscularly (Figure 1). The Beta-Ac was a gift from Merck Sharp & Dohme Corp. as a preparation of Beta-Ac that is equivalent to that in Celestone; a 0.25-mg/kg dose approximates the clinical dose of 12 mg of betamethasone for a 50 kg woman and was the same dose used for our previous studies.^9^, ^14^

For delivery, pregnant ewes were anesthetized with intravenous midazolam (0.5 mg/kg) and ketamine (10 mg/kg), followed by spinal anesthesia with 3 mL of 2% (20 mg/mL) lidocaine 48 hours after the first intramuscular treatment injection between 121 and 123 days of gestation. The head of the fetus was exposed through abdominal and uterine incisions; the fetal skin was infiltrated with lidocaine, and a tracheostomy was performed for insertion of an endotracheal tube. After delivery, fetuses were weighted, dried, and placed on a radiant warming bed (Cozy Cot; Fisher & Paykel Healthcare, Auckland, New Zealand) and covered with a plastic wrap for temperature control (Neowrap; Fisher & Paykel Healthcare) for ventilation.

### Mechanical ventilation

Mechanical ventilation (Fabian HFO; Accutronic Medical Systems AG, Hirzel, Switzerland) was started immediately with the intermittent positive pressure ventilation mode with standardized settings: initial peak inspiratory pressure (PIP) of 40 cm H_2_O, positive end expiratory pressure of 5 cm H_2_O, respiratory rate of 50 breaths per minute, inspiratory time of 0.5 seconds, and 100% heated and humidified oxygen. Animals were kept sedated with intramuscular ketamine to avoid spontaneous breathing. We inserted an umbilical artery catheter for blood sampling. Tidal volume (V_t_) was measured continuously and kept between 8.5 and 9.5 mL/kg by adjustment of the positive inspiratory pressure only, but with the maximal pressure limited to 40 cm H_2_O. We measured temperature, blood pressure, ventilator data (PIP, V_t_, and compliance), and performed blood gas measurements at 10, 20, and 30 minutes of ventilation. Dynamic compliance was recorded from the ventilator. The ventilation efficiency index, an integrated assessment of ventilation and gas exchange, was calculated with the use of the formula ventilation efficiency index = 3800/ respiratory rate (PIP–positive end expiratory pressure) × Pco_2_ (mm Hg).^15^

After 30 minutes of ventilation, lambs were disconnected from the ventilator, and the endotracheal tube was clamped for 2 minutes to achieve complete atelectasis by oxygen absorption. Lambs were euthanized with pentobarbital and weighed; the chest was opened for visual evaluation of gross lung injury as pulmonary hemorrhage, pulmonary interstitial emphysema, gas pockets within the lung, or subpleural dissection of gas. A pressure-volume curve was measured with air inflation of the lungs to a pressure of 40 cm H_2_O followed by deflation.^9^ Lung samples were snap frozen for molecular analysis.

### Messenger RNA quantitation by quantitative polymerase chain reaction

Frozen lung samples were homogenized, and total RNA was isolated with TRIzol (Invitrogen, Carlsbad, CA). Reverse transcription was performed with the Verso cDNA kit (Thermo Scientific, Waltham, MA) to produce single-strand complementary DNA. Amplification was performed with sheep-specific primers with Taqman probes (Applied Biosystems, Foster City, CA) for the following genes that are associated with fetal lung maturation: surfactant protein A (SFTPA), surfactant protein B (SFTPB), surfactant protein C (SFTPC), surfactant protein D, ATP-binding cassette subfamily A member 3 (ABCA3), and aquaporin 5 (AQP5). The messenger RNA (mRNA) expression for each gene was normalized to the mRNA for the ribosomal protein 18s as internal standard. Final data are expressed as fold increase over the mean control value for animals that were treated with intramuscular saline solution.

### Pharmacokinetics

For the pharmacokinetics studies, time-mated ewes had recovery surgery for placement of double lumen maternal jugular and single lumen fetal jugular catheters at 114–116 days of gestation as described previously.^16^ Animals were allowed to recover for 48 hours and then were assigned to receive intramuscular treatments of Beta-P 0.125 mg/kg, Beta-Ac 0.125 mg/kg, or Beta-Ac 0.5 mg/kg. We chose the dose of Beta-P of 0.125 mg/kg because this is the amount of Beta-P in a dose of Beta-P+Beta-Ac 0.25 mg/kg. Each animal received a second intramuscular injection 48 hours later to allow for drug clearance after the first treatment and before a second injection. Maternal and fetal blood samples of 2 mL were collected 10 minutes before and then 1, 2, 3, 4, 6, 8, 10, 12, 14, and 24 hours after intramuscular injection into chilled K_3_EDTA vacutainers. The blood was centrifuged at 3000*g*, and the plasma was frozen at –80°C for betamethasone concentration measurements. Maternal and fetal plasma samples and betamethasone standards (500, 250, 100, 50, 25, 12, and 0 ng/mL) in control fetal sheep plasma were extracted as previously described and analyzed by mass spectrometry.^13^ The limit of the detection of this assay is 1 ng/mL, and a cutoff >30% of the limit was used as a zero point for analysis. Data were fitted to a 1-compartment model with PKSolver. All *R*^2^ values for calibration curves were >0.98.

### Statistical analysis

Data are presented as bar graphs of the mean and standard deviations with discrete data points for each animal. Sample sizes were calculated with the use of our previous data in which control animals had a mean V40 of 8 mL/kg with a standard deviation of 3.5 and in which treated animals had a mean V40 of 16 mL/kg. With alpha set at .05 and power of 0.8, the sample size that was needed to identify differences between groups was 8. Considering loss of animals because of preterm labor, we planned for 10 animals per group. Statistical tests were performed with Prism software (GraphPad Software Inc, San Diego, CA). Initial comparisons were performed with analysis of variance, followed by multiple groups’ comparison with Holm-Sidak’s post-hoc test to compare control animals vs each treatment group, and the standard treatment (Beta-Ac+Beta-P 0.25 mg/kg × 2) vs other treatment groups. Significance was attributed for probability values <.05.

## Results

Fifty-nine animals completed the protocols. One animal in the Beta-Ac+Beta-P group was excluded because of illness and preterm labor before receiving any treatment. No animals had signs of preterm labor after ANS treatment. Groups had similar baseline characteristics that included gestational age, sex distribution, birthweight, and cord blood gas values (Table 1). Vital signs did not significantly differ after 30 minutes of ventilation (data not shown).

**Table 1:** Summary of animal data for ventilation study

Variable	Control	Betamethasone-acetatec+betamethasone-phosphate		Betamethasone-acetate×1		
		0.25 mg/kg ×2	0.25 mg/kg ×1	0.5 mg/kg	0.25 mg/kg	0.125 mg/kg
Animals, n	10	9	10	10	10	10
Gestational age, d^a^	122±0.5	122±0.7	122±0.7	122±0.7	122±0.5	122±0.5
Weight, kg^a^	2.7±0.2	2.5±0.3	2.6±0.3	2.8±0.4	2.6±0.3	2.7±0.3
Sex ratio (male/female), n	8/2	6/3	6/4	4/6	6/4	7/3
Cord blood gas^a^						
pH	7.34±0.03	7.34±0.07	7.33±0.05	7.28±0.14	7.34±0.02	7.35±0.04
pCO_2_ (mm Hg)	51±3	51±5	50±5	59±21.2	47±3.5	51±4.2
pO_2_ (mm Hg)	18±2	17±2	18±3	15±2.4	16±2.7	17±2.7

*Schmidt et al. Betamethasone-acetate for fetal lung maturation. Am J Obstet Gynecol 2018*.

a Data are given as mean±standard deviation.

### Assessment of lung function

**Figure 2:**
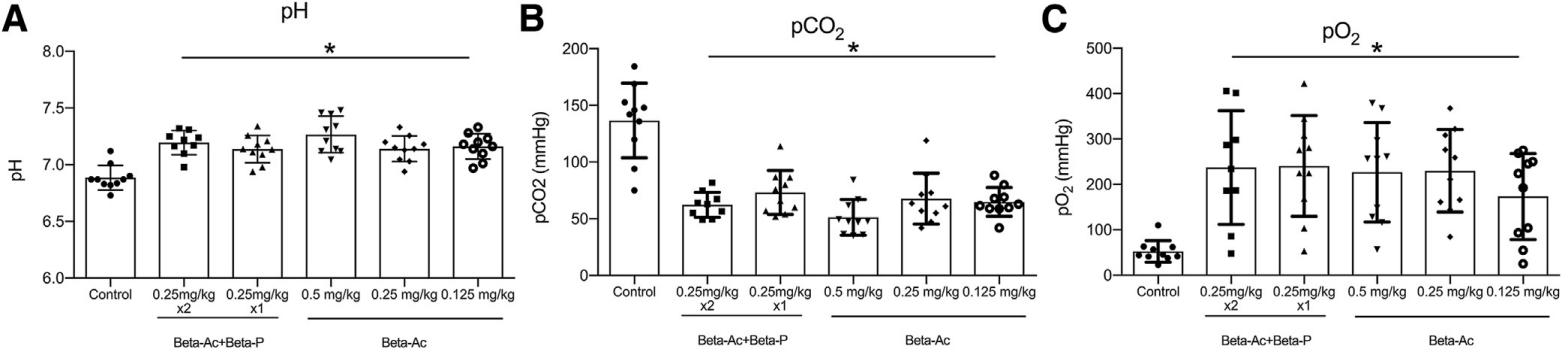
Blood gas measurements Blood gas measurements at 30 minutes of ventilation of preterm lambs. Betamethasone at all doses **A,** increased pH, **B,** decreased pCO_2_, and **C,** increased pO_2_. The *asterisk* indicates a probability value of <.05. *Beta-Ac+Beta-P*, betamethasone-acetate+betamethasone-phosphate. *Schmidt et al. Betamethasone-acetate for fetal lung maturation. Am J Obstet Gynecol 2018*.

**Figure 3:**
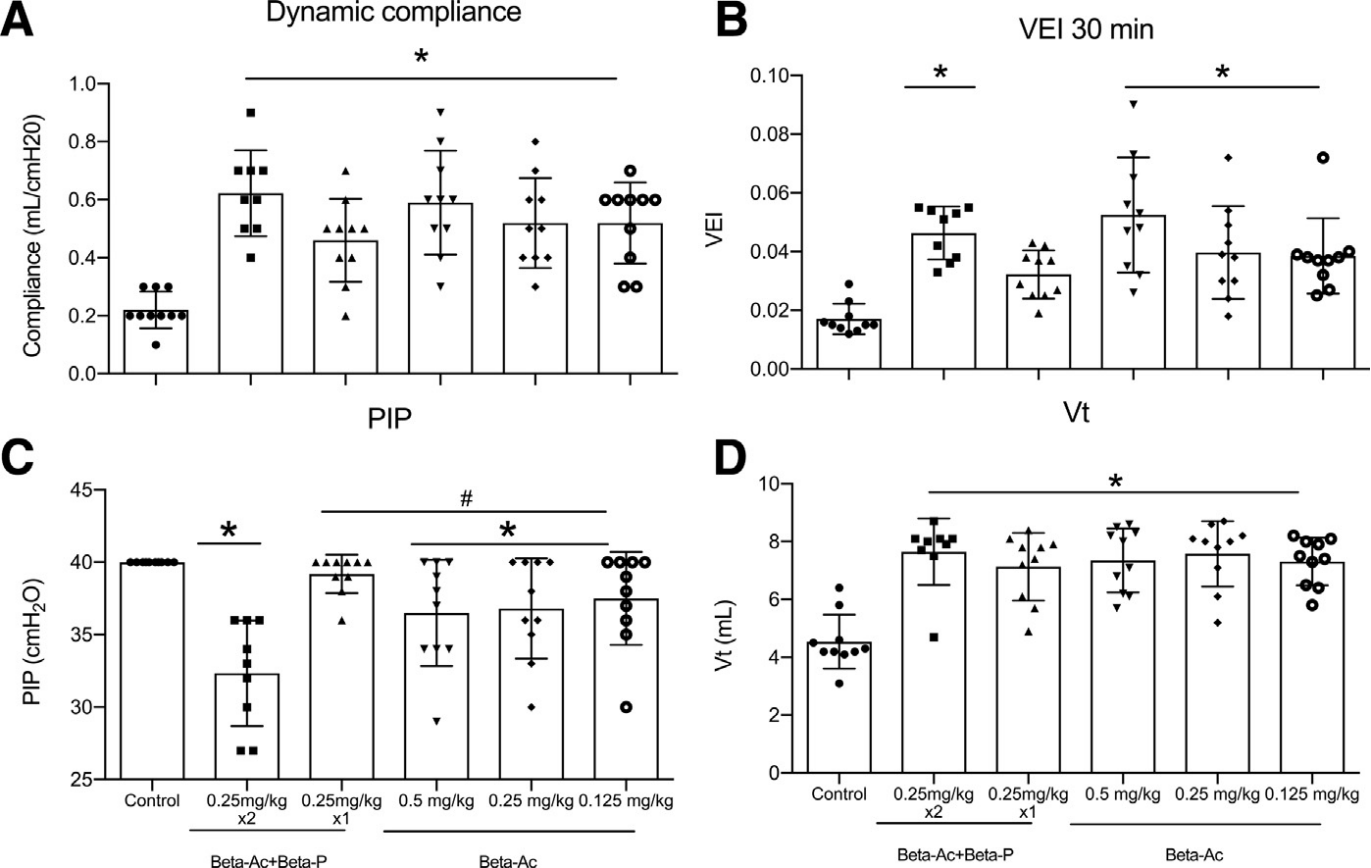
Ventilatory and lung mechanics variables Ventilatory and lung mechanics variables at 30 minutes of ventilation. **A,** Dynamic compliance; **B,** ventilatory efficiency index; **C,** positive inspiratory pressure; **D,** tidal volume. Any dose of betamethasone-acetate, which included the lowest dosage of 0.125 mg/kg, improved all measurements compared with control animals. The *asterisk* indicates a probability value of <.05. *Beta-Ac+Beta-P*, betamethasone-acetate+betamethasone-phosphate; *PIP*, positive inspiratory pressure; *VEI*, ventilator efficiency index; *V*_*t*_, tidal volume. *Schmidt et al. Betamethasone-acetate for fetal lung maturation. Am J Obstet Gynecol 2018*.

**Figure 4:**
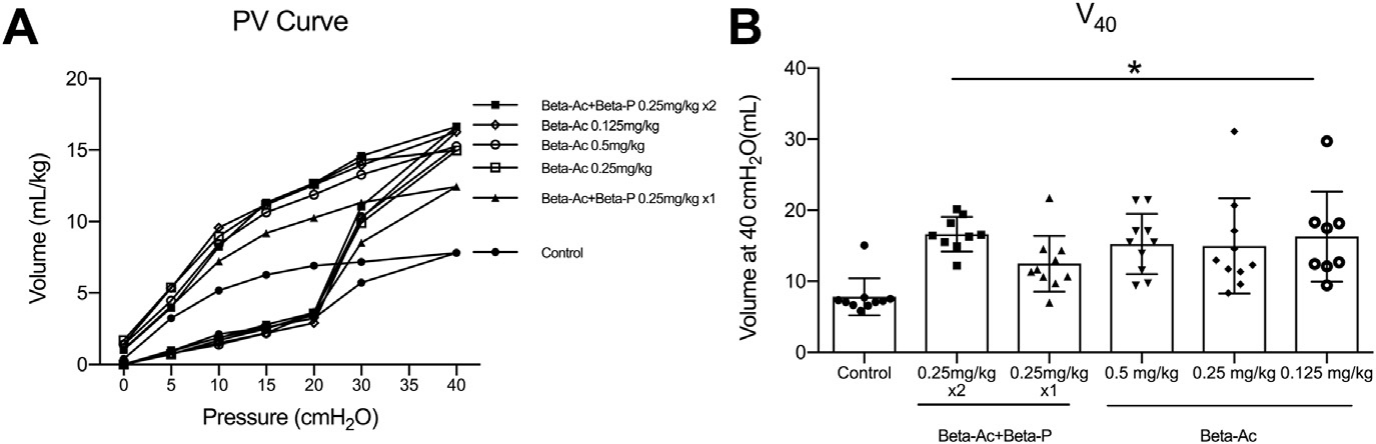
Static lung compliance of exteriorized lungs **A,** Pressure volume curves; **B,** volume at 40 cm H_2_O improved with any dose of Betamethasone-acetate. The *asterisk* indicates a probability value of <.05. *Beta-Ac+Beta-P*, betamethasone-acetate+betamethasone-phosphate; *PV*, pressure-volume; *V*_*40*_, volume at 40 cm H_2_O. *Schmidt et al. Betamethasone-acetate for fetal lung maturation. Am J Obstet Gynecol 2018*.

After 30 minutes of ventilation, treatment with any ANS dose improved blood gas measurements and dynamic lung compliance relative to control animals (Figures 2 and 3). The dynamic compliance increased from a mean of 0.2 ± 0.06 in control animals to 0.5 ± 0.15 for treated animals (*P*<.01). ANS treatment also improved ventilation efficiency index that showed better gas exchange compared with control animals, except for the group that was treated with 1 dose of Beta-Ac+Beta-P in which the difference was not statistically significant (unadjusted *P*= .03; adjusted for multiple comparisons *P*=.052). At 30 minutes, the PIP was lower in animals that were treated with Beta-Ac+Beta-P 0.25 mg/kg × 2, Beta-Ac 0.5 mg/kg or 0.25mg/kg compared with control animals, with the PIP that was required by the Beta-Ac+Beta-P 0.25 mg/kg × 2 being significantly lower compared with other treatment groups (*P*<.01; Figure 3). It is important to note that all animals in the control group required the maximal PIP of 40 cm H_2_O, which was not sufficient to achieve the target V_t_. All ANS treatment groups had similar V_t_ means at 30 minutes. Treatment with ANS also significantly improved static lung compliance compared with control animals as demonstrated by the pressure-volume curves and volume at 40 cm H_2_O (*P*<.01), except for animals that were treated with Beta-Ac+Beta-P 0.25 mg/kg × 1 (Figure 4). Gross lung injury was present in 3 animals (30%) in the control group, 1 animal (11%) in the Beta-Ac+Beta-P x 2 group, 4 animals (40%) in the Beta-Ac+Beta-P × 1 group, 2 animals (20%) in Beta-Ac 0.5 mg/kg group, 2 animals (20%) in Beta-Ac 0.25 mg/kg group, and 1 animal (10%) in Beta-Ac 0.125 mg/kg group. The frequency of gross lung injury was statistically similar in treatment groups compared with control animals.

### MRNA quantitation of lung maturation markers

**Figure 5:**
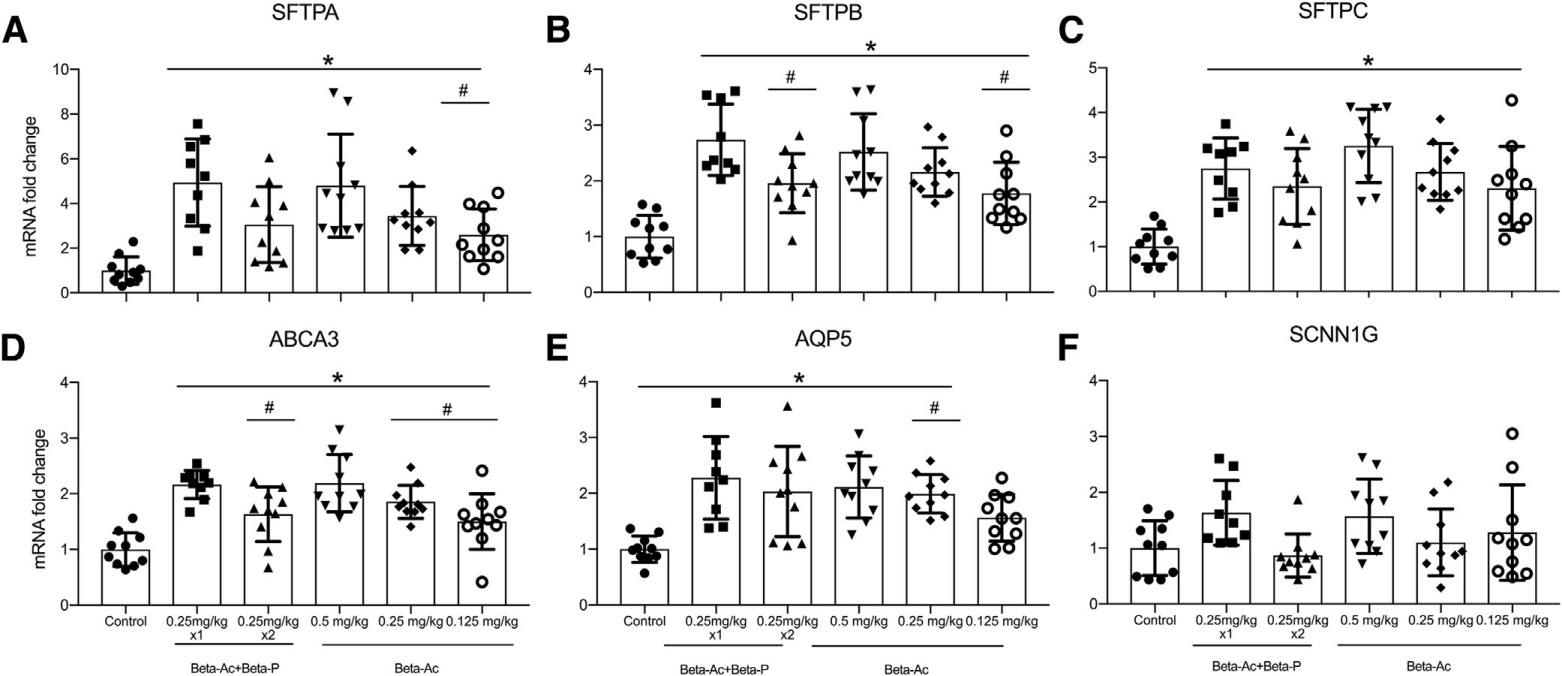
Messenger RNA quantitation in fold change relative to control animals **A,** surfactant protein A; **B,** surfactant protein B; **C,** surfactant protein C; **D,** ATP-binding cassette family A member 3; **E,** Aquaporin 5; **F,** Sodium channel epithelium 1 gamma subunit. The asterisk indicates a probability value of <.05. Single intramuscular dose of Beta-Ac increased the expression of surfactant proteins A, B, and C, ATP-binding cassette family A member 3, and Aquaporin 5. Surfactant protein D and sodium channel epithelium 1 gamma subunit expression were not changed by any treatment. *ABCA3*, ATP-binding cassette family A member 3; *AQP5*, Aquaporin 5; *Beta-Ac*, Betamethasone-acetate; *Beta-Ac+Beta-P*, betamethasone-acetate+betamethasone-phosphate; *mRNA*, messenger RNA; *SCNN1G*, sodium channel epithelium 1 gamma subunit; *SFTPA*, surfactant protein; *SFTPB*, surfactant protein B; *SFTPC*, surfactant protein C. *Schmidt et al. Betamethasone-acetate for fetal lung maturation. Am J Obstet Gynecol 2018*.

All ANS treatments increased mRNA expression of SFTPA, SFTPB, SFTPC, ABCA3, and AQP5, compared with control animals (Figure 5). Both the 1-dose Beta-Ac+Beta-P and the Beta-Ac 0.125 mg/kg treatments resulted in lower mRNA quantity of SFTPB, ABCA3, and AQP5, compared with the 2 doses of Beta-Ac+Beta-P 0.25 mg/kg. Compared with the 1-dose Beta-Ac+Beta-P, the Beta-Ac 0.125 mg/kg treatment had lower mRNA quantity of SFTPA (*P*=.01), SFTPB (*p*<.01), ABCA3 (*P*<.01), and AQP5 (*P*=.03). There were no differences between the 1-dose Beta-Ac+Beta-P and Beta-Ac 0.25 mg/kg or 0.5 mg/kg. ANS treatment did not increase mRNA expression of SFPTD or sodium channel epithelium 1 gamma subunit.

### Pharmacokinetics

**Figure 6:**
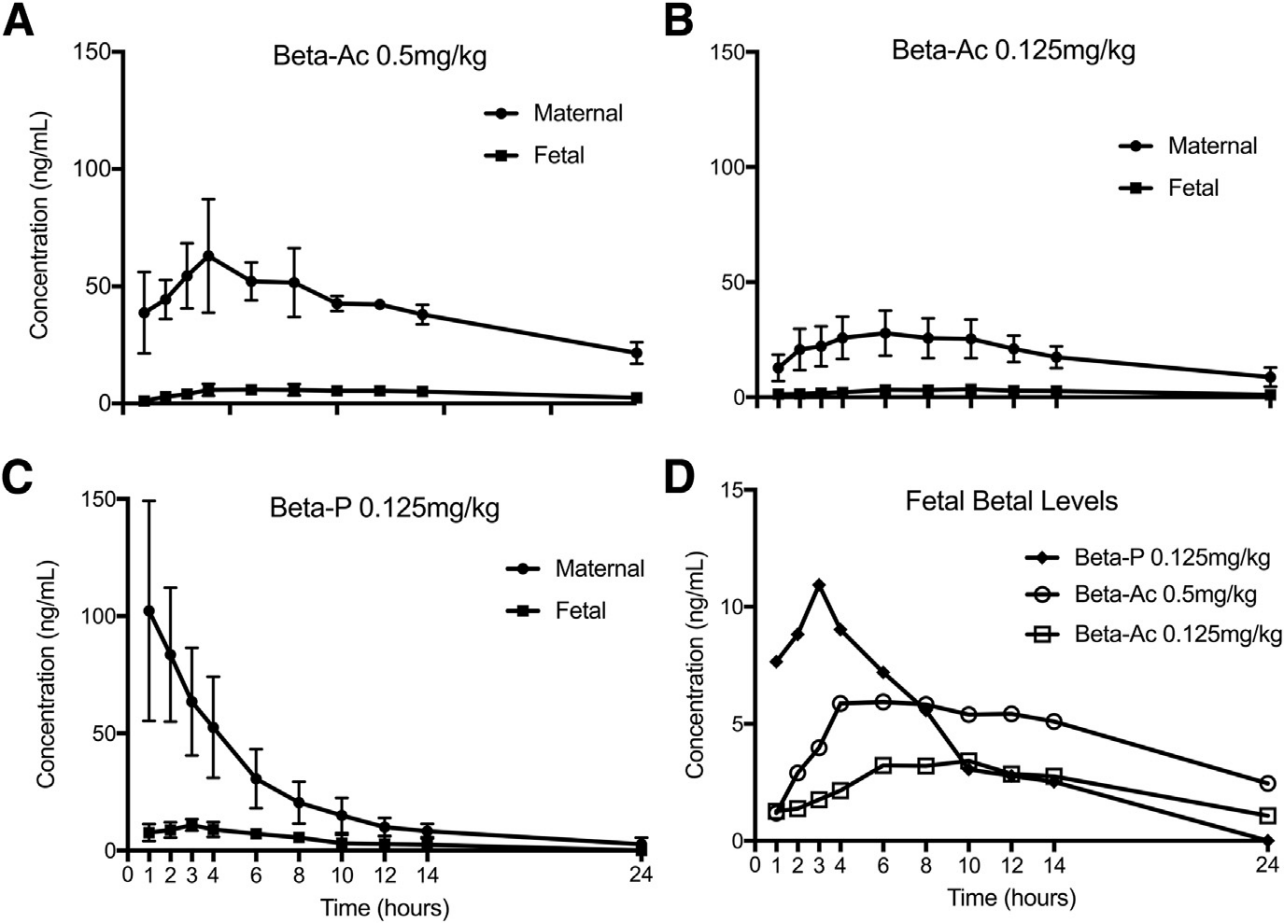
Average plasma concentrations of betamethasone after maternal intramuscular administration of antenatal steroids **A,** Maternal (*closed circle*) and fetal (*closed square*) plasma concentrations of betamethasone after 0.5 mg/kg of betamethasone-acetate had a maternal peak concentration of 57 ng/mL at 4 hours and fetal peak concentration of 6 ng/mL at 8 hours. **B,** Maternal (*closed circle*) and fetal (*closed square*) plasma concentration of betamethasone after 0.125 mg/kg of betamethasone-acetate had a maternal peak concentration of 27.6 ng/mL at 5.6 hours and fetal peak concentration of 3.1 ng/mL at 8.5 hours after intramuscular injection. **C,** Maternal (*closed circle*) and fetal (*closed square*) plasma concentrations of betamethasone after 0.125 mg/kg of betamethasone-phosphate had a maternal peak concentration of 102 ng/mL at <1 hour and fetal peak concentration 11 ng/mL at 2.4 hours after intramuscular injection. **D,** Comparison of fetal plasma concentrations for intramuscular betamethasone-phosphate 0.125 mg/kg (*close diamond*), betamethasone-acetate 0.5 mg/kg (*open circle*), and betamethasone-acetate 0.125 mg/kg (*open square*) had a lower peak fetal concentration for a similar total dose of antenatal steroids after intramuscular betamethasone-acetate compared with betamethasone-phosphate (3.1 vs 11, respectively; *P*<.05) and lower total fetal exposure measured by the area under the curve. *Beta-Ac*, betamethasone-acetate; *Beta-P*, betamethasone-phosphate. *Schmidt et al. Betamethasone-acetate for fetal lung maturation. Am J Obstet Gynecol 2018*.

Free betamethasone was detected in maternal and fetal plasma after Beta-Ac injections for the 24 hours, except for 1 fetus. After intramuscular Beta-P, betamethasone was not detected (<1 ng/mL) in the maternal plasma at 24 hours in 3 of 5 ewes and in none of the fetuses. In 2 of the 4 fetuses, betamethasone was not detected at and after 10 hours after the Beta-P injection. For a similar total dose of 0.125 mg/kg, Beta-Ac resulted in lower maximal maternal (27.6 vs 102 ng/mL) and fetal (3.1 vs 9.3 ng/mL) concentrations and total fetal exposure (area under the curve, 56 vs 78 ng/mL•h) than Beta-P (*P*=.04; Figure 6 and Table 2). The fetal concentration was approximately 10% of the maternal concentration for the 3 treatments. Total fetal exposure to ANS, which was measured by the area under the curve, was lower in Beta-Ac compared with a similar dose of Beta-P.

**Table 2:** Pharmacokinetic measurements of maternal and fetal plasma betamethasone after intramuscular injections of beta-phosphate or beta-acetate

Measurement	Maternal			Fetal		
	Beta-phosphate	Beta-acetate		Beta-phosphate	Beta-acetate	
	0.125 mg/kg	0.125 mg/kg	0.5 mg/kg	0.125 mg/kg	0.125 mg/kg	0.5 mg/kg
Half-life, h	2.4	9.2	11.9	5.1	6.2	6
Time of maximal concentration, h	0.87	5.6	4	2.4	8.5	7.9
Maximum serum concentration, ng/mL	102	27.6	57.4	9.3	3.1	6
Area under the curve 0-t, ng/mL•h	557	443	941	78	56	104

*0-t*, area under the curve from time 0 the the last time measured.

*Schmidt et al. Betamethasone-acetate for fetal lung maturation. Am J Obstet Gynecol 2018*.

A 4-fold increase in the administered dose of intramuscular Beta-Ac, from 0.125–0.5 mg/kg resulted in approximately a 2-fold increase in the mean maximal maternal and fetal concentration (maximum serum concentration, 27.6 vs 57.4 and 3.1 vs 6 mg/mL, respectively) and in the total fetal and maternal exposure (area under the curve, 443 vs 941 and 56 vs 104 ng/mL•h, respectively).

## Comment

We demonstrate that a single weight-based maternal dose of intramuscular Beta-Ac effectively induces physiologic and biochemical fetal lung maturation at lower doses than the standard of care treatment with Beta-Ac+Beta-P. The Beta-Ac results in decreased fetal exposure to corticosteroids compared with the Beta-P component of the clinically used combination. Corticosteroid toxicity in general results from high dose and long-term exposures, primarily when used for control animals of inclammation. The indication with ANS is for maturational signaling, which results from a prolonged (<24 h) low plasma level fetal exposure that can be achieved with Beta-Ac alone, which avoids the 2 high peak exposures from Beta-P. The other commonly used treatment of 4 doses of 6 mg of dexamethasone-phosphate will expose to fetus to 4 unnecessary high concentration exposures to dexamethasone.^17^

There have been no short-term adverse effects of ANS identified by metaanalysis of the randomized controlled trials, except for an increase in hypoglycemia from ANS exposure in infants who were delivered between 34 and 37 weeks.^18^ However, most trials were performed in high-income countries with availability of neonatal intensive care. A multicenter randomized trial in low- and middle-income countries found that ANS not only did not decrease mortality rates for infants who were born with a birthweight <5th percentile but it also increased neonatal mortality rates for all infants who were exposed to ANS prenatally, which could not be attributed to other components of the treatment intervention.^19^, ^20^

There remain concerns for long-term side-effects from fetal exposure to glucocorticoids, particularly neurologic, metabolic, and cardiovascular changes in later life.^21^, ^22^, ^23^ A limitation to the understanding the long-term effects is limited data from randomized trials. The original trials on ANS included preterm infants that were larger and of a more advanced gestational age than the current population of preterm infants.^4^ In a 30-year follow-up of former preterm infants who were included in a randomized clinical trial of ANS, Dalziel et al^24^ found increased insulin levels in glucose tolerance tests among subjects who were exposed to prenatal betamethasone, which suggests modulation of insulin sensitivity by ANS.

Because very preterm infants have a survival benefit with ANS, it would be unethical to perform new trials to assess for long-term outcomes in this population. Hence, observational studies have attempted to address potential side-effects of ANS. Very preterm newborn infants who were exposed to betamethasone have lower birthweights, lengths, and head circumferences than matched term-born control infants or infants who were exposed to preterm labor but not treated with ANS who were born at term.^25^, ^26^ ANS also affects neonatal basal cortisol levels and response to stress. In the neonatal period, prenatal exposure to multiple courses of ANS are associated with lower cortisol levels at baseline and after stress compared with single courses.^27^, ^28^ At school age, a history of prenatal exposure to a single course of betamethasone was associated with increased cortisol response to a stress compared with control infants.^22^ Similar effects of ANS on fetal growth and long-term metabolism have been reported in multiple experimental studies of fetal exposure to glucocorticoids; other investigators have found no or beneficial effects of ANS.^29^, ^30^, ^31^ Given these concerns, dosing optimization with minimization of fetal exposure could prevent or decrease long-term side-effects. These varied observations are not based on randomized populations.

There has been no optimization of ANS for fetal lung maturation. A Cochrane review compared maternal and neonatal outcomes with different treatments and found no differences in neonatal respiratory outcomes.^3^ This metaanalysis included 12 clinical trials with 7 different treatment strategies, which underscored the wide variation in practice. A randomized controlled trial is a costly and imprecise way to test drug doses. The preterm lamb model can be used as an excellent translational model to test drug dosing as preterm fetal lung maturation and neonatal ventilation because of the similarities in fetal lung development between sheep and humans. Fetal sheep are also used extensively to study maternal-fetal interactions because of the ability to place chronic maternal and fetal catheters.^32^, ^33^ Limitation of the model is the inability to directly assess ANS effects on other neonatal complications such as necrotizing enterocolitis, intraventricular hemorrhage, or long-term outcome. In this study, a single dose of 0.125 mg/kg of Beta-Ac resulted in similar improvements in lung compliance, ventilation efficiency, and gas exchange as the clinically used Beta-Ac+Beta-P regimen, with decreased fetal exposure to corticosteroids. Even though the PIP at 30 minutes was not significantly different for the lowest dose of Beta-Ac compared with control animals, this may have resulted from the limiting of the PIP to avoid air leaks. Only 4 animals in the Beta-Ac 0.125 mg/kg group required the maximal PIP of 40 cm H_2_O with V_t_ range of 5.8–7.5 mL/kg among those animals; all animals in the control animals group required the maximal PIP with a V_t_ range of 3.1–6.4 mL/kg (mean, 4.6 mL/kg). Both dynamic and static lung compliance were improved in the animals that were treated with Beta-Ac 0.125 mg/kg and were similar to the standard dosing. A dose <0.125 mg/kg should be tested for efficacy.

The lowest Beta-Ac dosing also increased mRNA expression levels of surfactant proteins A, B, and C, ABCA3, and AQP5 compared with control animals. The mRNA expression of surfactant proteins A and B, ABCA3, and AQP5 was significantly higher in the animals that received 2 doses Beta-Ac+Beta-P, compared with the lowest dose of Beta-Ac. We did not perform protein concentration measurements because protein differences will not occur until 4–5 days after ANS administration in previous studies.^34^ Given that these 2 groups had similar lung compliance and gas exchange, this difference may not be biologically relevant.

The pharmacokinetic findings regarding peak concentrations, half-life, and maternal-fetal transfer are in accordance with previous pharmacologic studies.^9^, ^10^, ^13^ Given that the Beta-Ac component is only available commercially in combination with Beta-P, the pharmacologic properties of Beta-Ac alone have not been explored thoroughly. Jobe et al^9^ previously reported the pharmacokinetics of the 0.25 mg/kg of Beta-Ac alone in 3 pregnant sheep. In our study, a single 0.125-mg/kg intramuscular dose of Beta-Ac improved lung mechanics and gas exchange to levels similar to the standard 2-dose regimen of Beta-Ac+Beta-P, which provides 0.25 mg/kg of Beta-P and 0.25 mg/kg of Beta-Ac. Maternal exposure was also reduced significantly with Beta-Ac compared with Beta-P. The Australasian Randomized Trial to Evaluate the Role of Maternal Intramuscular Dexamethasone trial^35^ currently is comparing 2 doses given 24 hours apart of 12 mg of Beta-Ac+Beta-P to 2 doses of 12 mg of dexamethasone-phosphate, which will yield even higher fetal plasma dexamethasone exposures. The results of this trial will provide more information on the differences between treatments.

Considering the possible long-term effects of ANS to the fetus, optimization of the dose with minimization of exposure is desirable. These concerns are amplified if ANS becomes used more widely for women who are at risk of preterm delivery in low resource environments, for previable fetuses who are at risk of preterm delivery, for women who deliver between 34 and 37 weeks gestational age, and for women who deliver by elective cesarean delivery at term.^18^, ^36^, ^37^ We demonstrate that a single dose of Beta-Ac may be an effective alternative to promote fetal lung maturation in women who are at risk of preterm delivery while minimizing maternal and fetal exposure to corticosteroids. These experimental evaluations of ANS provide a pathway to the clinical testing of new dosing strategies for ANS.
